# Machine learning to reveal hidden risk combinations for the trajectory of posttraumatic stress disorder symptoms

**DOI:** 10.1038/s41598-020-78966-z

**Published:** 2020-12-10

**Authors:** Yuta Takahashi, Kazuki Yoshizoe, Masao Ueki, Gen Tamiya, Yu Zhiqian, Yusuke Utsumi, Atsushi Sakuma, Koji Tsuda, Atsushi Hozawa, Ichiro Tsuji, Hiroaki Tomita

**Affiliations:** 1 Graduate School of Medicine, Tohoku University, Sendai, 980-0872 Japan; 2 Tohoku Medical Megabank Organization, Tohoku University, Sendai, 980-8573 Japan; 3 International Research Institute of Disaster Science, Tohoku University, Sendai, 980-8572 Japan; 4 RIKEN Center for Advanced Intelligence Project, Tokyo, 103-0027 Japan; 5 Department of Computational Biology and Medical Sciences, Graduate School of Frontier Sciences, The University of Tokyo, Chiba, 277-8568 Japan

**Keywords:** Computational biology and bioinformatics, Psychology, Environmental social sciences, Health care, Risk factors, Signs and symptoms, Mathematics and computing

## Abstract

The nature of the recovery process of posttraumatic stress disorder (PTSD) symptoms is multifactorial. The Massive Parallel Limitless-Arity Multiple-testing Procedure (MP-LAMP), which was developed to detect significant combinational risk factors comprehensively, was utilized to reveal hidden combinational risk factors to explain the long-term trajectory of the PTSD symptoms. In 624 population-based subjects severely affected by the Great East Japan Earthquake, 61 potential risk factors encompassing sociodemographics, lifestyle, and traumatic experiences were analyzed by MP-LAMP regarding combinational associations with the trajectory of PTSD symptoms, as evaluated by the Impact of Event Scale-Revised score after eight years adjusted by the baseline score. The comprehensive combinational analysis detected 56 significant combinational risk factors, including 15 independent variables, although the conventional bivariate analysis between single risk factors and the trajectory detected no significant risk factors. The strongest association was observed with the combination of short resting time, short walking time, unemployment, and evacuation without preparation (adjusted *P* value = 2.2 × 10^−4^, and raw *P* value = 3.1 × 10^−9^). Although short resting time had no association with the poor trajectory, it had a significant interaction with short walking time (*P* value = 1.2 × 10^−3^), which was further strengthened by the other two components (*P* value = 9.7 × 10^−5^). Likewise, components that were not associated with a poor trajectory in bivariate analysis were included in every observed significant risk combination due to their interactions with other components. Comprehensive combination detection by MP-LAMP is essential for explaining multifactorial psychiatric symptoms by revealing the hidden combinations of risk factors.

## Introduction

The symptoms of posttraumatic stress disorder (PTSD) after the disasters could take multiple trajectories^1^. In a population-based longitudinal study, Welch et al.^2^ identified six clusters of PTSD symptom trajectories after the disaster: low-stable (48.9%), moderate-stable (28.3%), moderate-increasing (8.2%), high-stable (6.0%), high-decreasing (6.6%), and very high-stable (2.0%).

Although factors that modulate the prognosis of PTSD symptoms after disaster have been investigated, the effect size of each single factor seems to be too weak to explain the variety of trajectories observed in clinical practice^3,4^. For example, Kessler et al.^3^ reported that middle age and low income were slightly associated with the trends in PTSD symptoms in a longitudinal surveillance study after a hurricane, but these risks explained only 2.1% of the variance in the trajectories of PTSD symptoms. Both Kessler et al.^3^ and Adams and Boscarino^4^ reported that the degree of exposure to stressful events was a significant predictor only for the onset of PTSD and not for the trends in PTSD after a disaster.

Considering the multifactorial nature of the condition, the most straightforward approach for obtaining useful information with sufficient effect sizes regarding the prognosis of PTSD would be effective accumulation of risk factors by considering interaction among the factors. Several studies have demonstrated interactions among risk and protective factors for the prognosis of PTSD symptoms^5–9^. Satisfaction with social support has a significantly larger positive effect on the prognosis of PTSD symptoms in females than in males^5^. Excessive alcohol intake can have a large impact on the exacerbation of PTSD symptoms in males^6^. Loss of family members or lack of family support influences the prognosis of PTSD more in younger subjects than in older subjects^7,8^. Drožđek et al. considers combinations of risk factors and shows the hidden long-term impacts of exposure to war and violence^9^.

In previous studies to elucidate combinational risk factors by focusing on interactions, the major limitation was that candidate risk factors were selected based on their association with the target symptom. However, a factor showing no association with the target symptom in bivariate analysis could plausibly contribute to reliable combinational risk predictors by strong interaction with other factors; such a risk factor that is apparent only in combinational analysis can be referred to as a “hidden risk component”. Therefore, although several risk predictors for PTSD prognosis have already been suggested by previous bivariate association studies, a comprehensive combination detection study based on a number of potential risk factors, without selection by other statistics, would be useful to detect reliable combinational risk predictors.

Despites the potential usefulness of comprehensive combination detection studies in detecting hidden risk components, such studies have been infeasible due to high computational costs and excessively severe multiple-testing correction. For example, if 30 potential risk factors were tested for combinational risks, there would be 2^30^ (> 10^9^) possible combinations. Therefore, if all of these combinations were tested, the computational cost would be so high as to render the calculation impractical, and the raw *P* value would need to be no greater than 4.6 × 10^−11^ for “significance” at the α = 0.05 level after a Bonferroni correction.

The Massive Parallel Limitless-Arity Multiple-testing Procedure (MP-LAMP) was developed to explore significant combinational risk factors among a large number of independent variables^10,11^. LAMP is a novel algorithm that renders comprehensive detection of significant combinations feasible by reducing computational costs and preventing excessively severe multiple-testing correction by avoiding unnecessary significance tests of potential risk combinations that (1) cannot be significant or (2) are completely dependent on each other^12–15^. First, if the number of subjects with a potential risk combination is sufficiently small, the association between the combination and the target variable (e.g., psychiatric symptom score) can never be significant, regardless of the values of the target variables (detailed in Supplementary methods). These combinations do not influence the familywise error rate^16^ and are ignored in the LAMP algorithm. Second, the possible risk combinations are often completely dependent on each other. For example, when all of the subjects with risk factors A and B have risk factor C, the subject group with the risk combination of A and B and the subject group with A, B, and C would be the same. In this case, LAMP conducts significance tests only for combinations with more components *(*i.e*.,* A, B, and C) and avoids unnecessary duplicate tests. Through the abovementioned two procedures, the LAMP algorithm makes comprehensive significant combination detection feasible under the condition that the familywise error rate is controlled rigorously under the threshold. MP-LAMP is a software tool to accelerate LAMP calculations and render it feasible in large datasets by utilizing parallel calculations.

The current study targets a relatively long-term prognosis of PTSD symptoms because of clinical importance. According to previous studies, the short-term prognosis of PTSD was largely explained by the severity of PTSD symptoms just after the disaster^3,4^. Then, people who have severe PTSD just after the disaster easily obtain access to specialized treatments. In contrast, the long-term prognosis of PTSD is weakly explained by the symptoms just after the disaster^17^, and appropriate support is possibly not provided to people who suffer from delayed PTSD symptoms. In this case, the prediction of PTSD prognosis based on various risk factors would be useful to provide adequate support to high-risk populations. Nevertheless, the long-term prognosis of PTSD after a natural disaster has rarely been surveyed, and there is little evidence we can consult^17^.

In the current study, we applied MP-LAMP to identify combinational risk factors that modulate the prognosis of residents severely affected by the Great East Japan Earthquake regarding PTSD symptoms, as measured by Impact of Event Scale-Revised (IES-R) scores. We conducted annual surveys to evaluate the mental health condition of all residents whose houses were located in the town of Shichigahama and had been destroyed or severely damaged by the catastrophe^18,19^. We utilized datasets including 624 subjects who completed the surveys in 2011, 2012 and 2018. To investigate the risk factors that modulate the prognosis of PTSD symptoms, we used IES-R scores in the 8th year adjusted for those in the 1st year, referred to hereinafter as “PTSD trajectory scores”, as the target variables, following the methods of previous studies^3,4,20^. The PTSD trajectory score represents the change in PTSD symptoms that is not explained by the baseline PTSD symptoms. This derived measure is beneficial in the search for useful risk factors that can be used in conjunction with baseline symptomatology to predict the prognosis of PTSD symptoms. We utilized MP-LAMP to explore combinational explanatory factors for PTSD trajectory scores based on information about stressors (experience related to the tsunami or earthquake, loss of loved ones), sociodemographics, lifestyle, and clinical information collected just after the disaster. The results of MP-LAMP regarding combinational risk factors were compared with those of the conventional association tests for individual risk factors, referred to hereinafter as “bivariate analyses”.

## Material and methods

### Subjects

This study is based on a health survey administered as part of a project called the Shichigahama Health Promotion Project^18,19^. The first survey was conducted in November 2011 following the Great East Japan Earthquake and Tsunami of March 11th, 2011. Annual surveys were conducted thereafter, and the latest survey before the current analysis was conducted in October 2018. This study is based on the questionnaire collected on the first, second, and eighth (i.e., the latest) survey. In the study population of 2,478 Japanese subjects who were at least 18 years old and whose houses were totally collapsed or severely damaged, 1,791 subjects participated in the first year survey and returned the questionnaire after giving written informed consent. Among those subjects, 1,173 participated in the second survey, and 636 participated in the first, second, and eighth surveys. Then, the subjects who omitted > 20% of items on the IES-R items or potential risk factors were excluded based on previous studies^21–23^, and those who omitted > 50% of items on the questionnaire were also excluded based on the literature reviewed^24–26^.

### Questionnaire

Because the purpose of the current study is to elucidate risk predictors available just after the disaster for the prognosis of PTSD, the data utilized as potential risk factors were mostly based on the questionnaire collected in the first year. The data from the first survey included sociodemographic characteristics (age, sex, and employment status), lifestyle (smoking status, alcohol drinking, daily time spent walking/sitting/sleeping), clinical information (past medical history), the Kessler Psychological Distress scale (K6), the Athens insomnia scale (AIS), and the Lubben Social Network Scale-6 (LSNS-6). In addition, the data related to experiences of the earthquake and tsunami (the evacuation, witnessing the tsunami, life-threatening experiences, witnessing threats to other people’s lives, death of family or friends) and changes in income or work volume were collected in the second year survey. The abovementioned 61 variables were utilized as potential risk factors for the prognosis of PTSD in the following analyses.

### Outcome measures

The IES-R score was used as an indicator of PTSD symptoms. The respondents were asked about their PTSD symptoms over the previous week based on 22 questions, to which they responded by selecting “extremely” (4 points), “quite a bit” (3 points), “moderately” (2 points), “a little bit” (1 points), or “not at all” (0 points). The total scores ranged from 0 to 88. IES-R scores correlate well with the criteria for PTSD in the Diagnostic and Statistical Manual of Mental Disorders (DSM), and IES-R is one of the most commonly used metrics of PTSD symptomatology^27,28^. To evaluate the long-term change in PTSD symptoms that was not explained by the baseline PTSD symptoms, we utilized the eighth-year IES-R adjusted by the first year IES-R as a target variable in the following analysis; we refer to this measure as the “PTSD trajectory score” throughout the manuscript.

### Statistical analyses

After the abovementioned exclusion of subjects with high rates of missing responses on the questionnaire, the missing rates among IES-R items and potential risk factors were 0.5% and 2.9%, respectively. After confirming that there were no statistically significant bias effects caused by the missing data, we imputed the missing numbers nonparametrically using the missForest package^29^ in R because the LAMP analyses require datasets without missing data (Supplementary methods).

To detect all significant combinational risk factors, we used MP-LAMP. MP-LAMP is a software package to accelerate the LAMP algorithm^10,11^. The LAMP algorithm renders combinational significance detection feasible by ignoring combinations that cannot be significant or are completely dependent on each other^12–14^. To select testable combinations, the LAMP algorithms utilized machine learning techniques of frequent itemset mining. The LAMP algorithm utilizes a calibrated Bonferroni method to correct for multiple testing under the condition that the familywise error rate is controlled rigorously under the threshold. The LAMP was originally developed for biological data, but the method has already been used for survey data^30,31^. In the current analysis, the main analysis was not adjusted for potential confounding factors following the previous LAMP-based survey studies^30,31^, while additional analysis adjusted for age and sex was also performed to check the consistency of the results. In this additional analysis, the PTSD trajectory score adjusted for age and sex was utilized as a target variable. The source code for MP-LAMP is available at https://github.com/tsudalab/mp-lamp.

Because the independent variables must be binary in order for MP-LAMP to detect combinational risks, some variables were converted to binary values by setting cutoffs. For those of the scales that already had proposed cutoffs, those cutoffs were utilized (5/6 and 12/13 for K6, 5/6 for AIS, and 11/12 for LSNS-6)^32–35^. For other ordinal variables with more than three levels and for all continuous variables, the variables were first discretized into ordinal variables with three levels of approximately equal frequency by using the infotheo R package^36^ and then converted into binary variables with the highest or lowest level as the risk group and the remaining two levels as the nonrisk group. This division was chosen because MP-LAMP requires substantially more computational time to analyze independent variables with a higher frequency of membership in the risk group. The detailed process of converting ordinal variables into binary variables is shown in the Supplementary methods.

For comparison with the results of the combinational analysis, conventional association analysis for the same response and independent variables was also performed. We implemented linear regression adjusted by age and sex to evaluate the association between adjusted IES-R and each independent variable, a procedure referred to as “bivariate analysis” throughout the manuscript in contrast to the combinational analysis by MP-LAMP. Multiple-testing correction was performed using the Bonferroni method to control the familywise error rate.

The Mann–Whitney U test was implemented to evaluate the association between the potential risk combinations and the PTSD trajectory score. In addition to the MP-LAMP software, R was utilized in statistical analyses^37^. *P* < 0.05 was considered to indicate statistical significance.

### Ethics approval and consent to participate

All protocols for the studies were approved by the Ethics Committee of Tohoku University. Written informed consent was obtained from all subjects. This study was carried out according to the principles expressed in the Declaration of Helsinki.

## Results

Of the variance of IES-R scores in the 8th year, only 23.5% was explained by the baseline IES-R, and the remaining explanatory factors were explored using the PTSD trajectory score as a target variable in the following analyses.

### Demographic and trauma-exposure information

The demographic characteristics and trauma exposure of the subjects are summarized in Table 1. Older age, female gender and a high degree of traumatic exposure had a strong association with high baseline IES-R scores; however, they had a weaker association or no association at all with the PTSD trajectory score. After correcting for multiple testing, there were no significant associations between PTSD trajectory scores and demographic or trauma information in bivariate analyses.

**Table 1:** Demographic characteristics and trauma exposure of participants.

	Number of subjects	IES-R score in the first year		*P* value^a^	IES-R score in the eighth year		PTSD trajectory score		*P* value^a^
		Mean	SD		Mean	SD	Mean	SD	
Total	624	20.0	15.2		11.5	13.7	0	12.0	
**Age**				3.3 × 10^−5^					7.2 × 10^−3^
< 30	63	15.3	12.5		5.9	8.9	− 3.64	10.4	
30–49	182	17.4	14.9		10.0	13.8	− 0.47	11.0	
50–69	260	20.7	14.4		11.8	12.7	− 0.05	11.1	
≥ 70	119	24.7	17.2		16.4	16.2	+ 2.76	15.4	
**Gender**				3.1 × 10^−4^					0.45
Male	266	17.4	15.0		10.0	13.2	− 0.46	12.0	
Female	358	21.9	15.1		12.7	14.1	+ 0.34	12.1	
**Working status**				0.79					0.44
Employed	402	19.0	14.8		10.4	12.7	− 0.70	11.2	
Unemployed/seeking work	222	21.8	15.8		13.6	15.3	+ 1.26	13.3	
**Current smoking status**				0.75					0.36
No smoking	480	20.9	15.1		12.3	13.9	+ 0.34	11.9	
1–19 cigarettes/day	67	16.7	14.3		10.7	14.8	+ 0.55	13.6	
20 cigarettes/day or more	77	17.0	16.2		7.6	11.3	− 2.62	11.1	
**Current alcohol consumption**				0.14					0.80
No. of drinks	377	20.0	15.2		11.7	14.0	+ 0.16	12.0	
≤ 1 *go*/day^b^	129	20.7	15.2		11.4	13.5	− 0.46	10.9	
> 1 *go*/day	118	18.8	15.5		11.1	13.2	+ 0.00	13.2	
**Chronic diseases**^**c**^				0.24					3.1 × 10^−2^
Yes	206	22.9	16.4		15.3	15.3	+ 2.48	14.1	
No	418	18.5	14.4		9.7	12.5	− 1.22	10.6	
**Threat of death**				2.7 × 10^−6^					1.9 × 10^−2^
Yes	342	22.4	16.4		13.6	15.2	+ 0.95	13.5	
No	282	17.0	13.1		9.1	11.3	− 1.15	9.8	
**Witnessed actual/threatened death of others**				5.4 × 10^−3^					0.41
Yes	116	22.1	17.0		12.9	15.6	0.39	14.1	
No	508	19.5	14.8		11.2	13.3	− 0.09	11.5	
**Intense fear, helplessness, or horror**				2.2 × 10^−7^					0.36
Extreme	359	22.9	15.8		13.3	14.7	0.51	12.7	
Moderate	201	17.0	13.5		9.8	11.9	− 0.42	11.2	
None/slight	64	12.6	12.6		6.8	11.6	− 1.52	10.2	
**Witnessing tsunami**				0.12					0.15
Did not witness	301	18.9	14.2		10.6	12.6	− 0.47	11.2	
Witnessed	300	20.8	16.1		11.9	14.0	− 0.02	12.5	
Swept by tsunami	23	22.6	16.2		19.2	20.9	6.51	15.5	
**Loss of family/friends**				6.1 × 10^−2^					0.58
Yes	298	21.6	15.4		12.8	14.6	0.55	12.7	
No	326	18.4	14.9		10.4	12.8	− 0.50	11.3	

*IES-R* Impact of Event Scale-Revised, *PTSD* posttraumatic stress disorder, *SD* standard deviation.

^a^*P* values were based on a linear regression model using IES-R in the first year or PTSD trajectory score as a response variable and adjusted by age and gender. Multiple testing was not corrected for.

^b^22.8 g of alcohol amounts to 1 *go* or traditional unit of sake (180 ml), which also approximates two glasses of wine (200 ml) or beer (500 ml) in terms of alcohol content.

^c^History of at least one of the following diseases: hypertension, diabetes, dyslipidemia, stroke, myocardial infarction, cancer, kidney disease, or liver disease.

### Comprehensive combinational risk detection analysis

**Figure 1:**
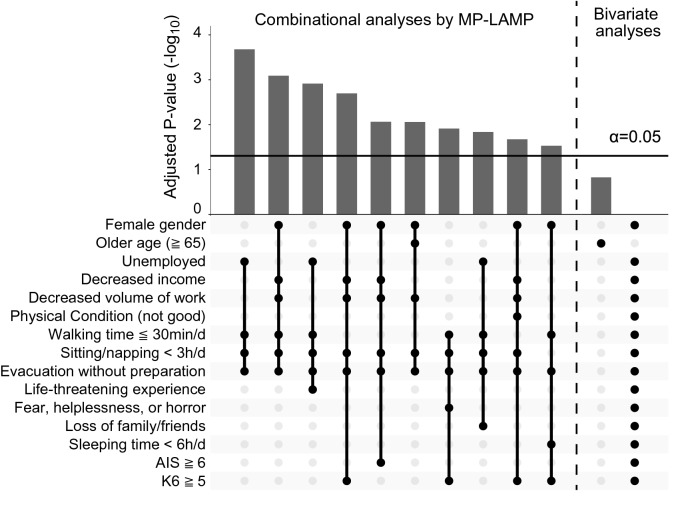
Adjusted *P* values of combinational/single risk factors significantly associated with a poor prognosis for PTSD symptoms. The Y-axis indicates the negative logarithms of the *P* values of combinational/single risk factor(s) significantly associated with IES-R trajectory scores and the *P* value of each component of the significant combinations. As a measure to control the familywise error rate by correcting for multiple comparisons, the *P* values for combinations were adjusted by MP-LAMP, and the *P* values for single factors were adjusted by the Bonferroni correction. Combinations are represented by points connected by lines, and single factors are represented by points without lines. Among 56 significantly associated combinations, the representative combinations (including the combinations whose *P* values were the smallest for each component) are shown. MP-LAMP substantially increased the power to detect significant predictors by testing combinations, in which the components of significant combinations were not necessarily associated with the target variable as individual risk factors.

The 61 abovementioned potential risk factors were subjected to comprehensive combinational risk detection analysis by MP-LAMP and bivariate analysis. Although bivariate analyses detected no significant predictors of PTSD trajectory scores, combinational association analyses by MP-LAMP detected 56 significant combinations, in which 15 independent variables were used at least once each as components. The *P* values of the representative significant combinations shown by MP-LAMP and the components of the significant combinations are illustrated in Fig. 1. Compared with bivariate analyses, the comprehensive combination detection approach substantially increased the power to detect significant predictors of PTSD trajectory scores. All of the significant combinations and the results of the bivariate analyses for individual risk factors are shown in Supplementary Tables S1 and S2.

The significant combinations yielded by comprehensive combination detection were completely different from the combinations selected solely based on the strength of association in the bivariate analyses, as the interactions among the risk factors also contributed to the strength of association in the combinational analysis. To maximize the association with the target variable through interactions among components, each significant risk combination identified by MP-LAMP included at least one component that had no association with the target variable (raw *P* value > 0.05) in bivariate analyses. The average (SD) numbers of interactions with *P* < 0.05 and *P* < 0.01 by analysis of variance among the components of the significant risk combinations were 4.9 (2.9) and 2.5 (1.3), respectively, which were substantially higher than the 95% confidence intervals of 1.2–1.9 and 0.3–0.7 calculated from randomly selected combinations consisting of the equivalent number of components (100,000 bootstrap replications; Supplementary Table S1).

The additional analysis adjusted for age and sex was also performed to check the consistency of the results, and the significant risk combinations in this analysis are shown in Supplementary Table S3. The significant risk combinations in this additional analysis largely overlapped with the main analysis. Specifically, the top 10 significant risk combinations in the main analysis were also significant in this additional analysis, while all 15 significant risk combinations in the additional analysis were also significant in the main analysis.

### The combination most strongly associated with the PTSD trajectory score

**Figure 2:**
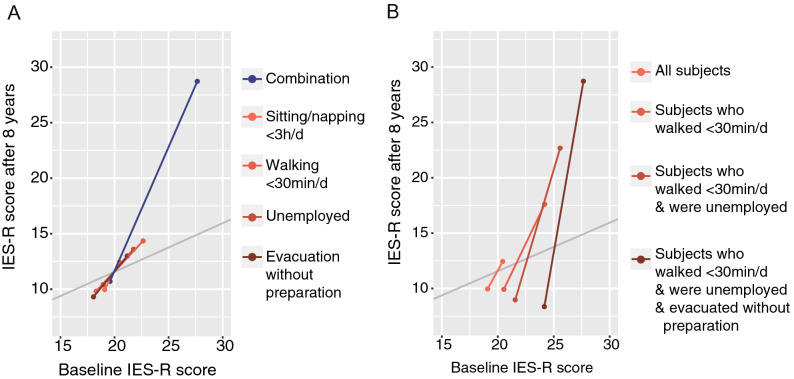
The effect size of the combination strongly associated with the change in PTSD symptoms on the IES-R scores at 1 year and 8 years after the event. Regarding the combination that had the strongest association with the poor trends of IES-R scores (i.e., unemployment, walking less than 30 min/day, sitting/napping less than 3 h/day, and evacuation without preparation), the effect sizes on the IES-R scores at baseline and follow-up are shown. The X- and Y-axes indicate the IES-R scores at 1 year and 8 years after the event, respectively. To illustrate the effect size of the risk factors on the IES-R scores, we illustrate the difference between the average IES-R scores of the risk group (the upper right points) and the scores of the nonrisk group (the lower left points) with points connected lines. The extended line in the direction of the Y-axis expresses a poor prognosis for IES-R scores by the risk factor. The regression line of the 8th-year IES-R score on the 1st-year IES-R score is shown in gray. (**A**) Comparison of the effect size of the combination and each component on the IES-R scores. (**B**) Evaluation of the interaction between short sitting/napping time and the other components by subgroup analysis.

The combination that was most strongly associated with the PTSD trajectory score was unemployment, walking less than 30 min/day, short resting time (sitting or napping for less than 3 h/day), and evacuation without preparation (adjusted *P* value = 2.2 × 10^−4^, and raw *P* value = 3.1 × 10^−9^). The effect size of this combination and its components on the IES-R scores are illustrated in Fig. 2A, and the combination was demonstrated to have a substantially stronger effect size on the IES-R in the 8th year than any single component.

Although short resting time was not significantly associated with the PTSD trajectory score in bivariate analyses (adjusted *P* value > 1, raw *P* value = 0.055), it had a significant interaction with short walking time (*P* value = 1.2 × 10^−3^), which was further strengthened by the other two components (*P* value = 9.7 × 10^−5^). To illustrate this significant interaction, Fig. 2B shows the interaction regarding effect sizes on IES-R scores between short sitting/napping time and the other components. The effect size of short sitting/napping time on the 8th-year IES-R score increased in the subgroup selected based on the other components, which reflected the interaction among these factors.

## Discussion

The current study used MP-LAMP to explore the combinational risk factors that modulate the long-term prognosis of PTSD symptoms after a disaster, showing that (1) the combinational risk approach increased the power and detected novel significant risk factors and that (2) the significant combinations detected by the comprehensive combination approach included interactions among the components.

Although bivariate analyses detected no significant risk factors, the combinational approach detected 56 combinational risk factors consisting of 15 independent variables, demonstrating that the combinational approach substantially increased the power to detect risk factors associated with PTSD trajectory scores. The remarkable point was not merely that the detection power was increased by the combinational analyses but that the risk factors newly detected in combinational analyses were completely different from the ones detected by loosening significance levels in the bivariate analyses. Among 15 independent variables included at least once in the significant combinations, there were 10 variables that had no association with the target variable in bivariate analyses (raw *P* value > 0.05); these 10 variables could be referred to as “hidden risk components”. Based on this finding, in the search for risk factors to increase predictive performance, the conventional approach of combining the previously reported risk factors would be useless for identifying the most reliable predictor combinations including hidden risk components; only a comprehensive combination detection approach considering all possible interactions among the variables, regardless of whether each variable would be counted as a risk factor based on bivariate analyses, could detect hidden risk components.

In the search for combinational predictors, the major reason to include hidden risk components that have no bivariate association with the target variable is that, although a factor may carry a low risk in bivariate analyses and lack a strong association with the target variable, it can interact with other components that increase the association between the combination and the target variable. The significant risk combinations detected by MP-LAMP consisted of the components among which there were significantly more and stronger interactions than randomly selected combinations. The interactions detected by analysis of variance included not only interactions among two components (49%) but also interactions among three or more components (51%). Most of the previous studies investigating interactions among risk factors for PTSD symptoms analyzed only the interactions between pairs of components among several risk factors^5,38^, mainly because comprehensive interactions including three or more components consist of an exponentially larger number of possible combinations. MP-LAMP resolved this problem by ignoring “untestable” combinations, whose frequency is too small to be significant, and investigated all possible interaction patterns without limitation of the number of components, which successfully revealed the significant risk combinations that explain the trajectory of PTSD symptoms.

The risk combination approach can provide useful interpretation for clinical practice based on the relationship between risk factors. Previous studies using conventional bivariate analysis stated that the degree of traumatic experience influenced only PTSD symptoms just after the disaster but did not influence PTSD symptom prognosis^3,4^. However, the results from the current risk combination analysis presented another view about the relationship between the traumatic experience and the prognosis of PTSD. In the current study, most of the significant risk combinations include the risk factors of a traumatic experience (e.g., evacuation without preparation or life-threatening experience), working status (e.g., unemployment), and lifestyle factors (e.g., short walking time or short resting time). The distribution of PTSD trajectory scores in the set of subjects selected by combinational or single risk factors is shown in Supplementary Fig. S1. As shown in this figure, although no single traumatic factor increased the PTSD trajectory score by itself, the combination of the traumatic factors, working status, and lifestyle factors increased the PTSD trajectory scores through the interactions. In clinical practice, these results imply that surveillance about not only the traumatic experience but also the social or lifestyle information is useful to assess the high-risk population for long-term prognosis.

In the current analyses, female gender was associated with elevated baseline PTSD symptoms (*P* value = 3.1 × 10^−4^) but did not influence the PTSD trajectory score (*P* value = 0.45) in bivariate analyses. However, the gender factor had a significant interaction with decreased income (*P* value = 2.7 × 10^−3^), physical condition (not good) (*P* value = 8.1 × 10^−3^) and older age (*P* value = 0.025), and was included in some of the significant risk combinations for PTSD trajectory scores. Based on these findings, the factor of gender alone cannot be considered to influence the trajectory of recovery from PTSD symptoms; however, the risks factors of income, physical condition, and age can influence recovery from PTSD symptoms more severely in females than in males.

The variance explained by the risk factors was calculated to compare the results with those of the previous studies (Supplementary Tables S1 and S2). Among the significant risk factor combinations, the combinations of unemployment, short walking time, short resting time, evacuation without preparation, life-threatening experience, and decreased income explained the largest variance in the PTSD trajectory score (8.5%). Among single risk factors, physical conditions (poor) and decreased work explained the largest variance (2.0%). The abovementioned values did not conflict with the findings of a previous study^3^. For example, Kessler et al. showed that the PTSD prognosis explained by the strongest risk factors (age and incomes) was 2.1% in a 2-year longitudinal surveillance study after a disaster^3^. The current study’s approach to creating risk combinations was shown to be useful to combine the effects of single risk factors.

The components of the significant risk combinations in the current study did not conflict with the previous PTSD prognosis study after the disaster^3,4^. The significant risk combinations in the current study were composed of gender, age, working condition, lifestyle factors (e.g., working time or sleeping time), life events (e.g., loss of family), and distress scale (i.e., K6 score). Although there are no risk combination studies, there are a couple of studies using bivariate analysis to search for risk factors for PTSD prognosis after the disaster. Kessler et al. performed a 2-year longitudinal study after Hurricane Katrina that suggested that PTSD prognosis was influenced by the risk factors of age and working condition^3^. Adams et al. performed a 2-year longitudinal study after the World Trade Center Disaster, which suggested that the change in PTSD symptoms was influenced by negative life events, Latino ethnicity, and reduced self-esteem^4^. Considering the similarity between the results of the current study and those of previous studies, the current results could be applied to PTSD prognosis after various types of disasters. In contrast, the risk factors for PTSD prognosis from the other types of trauma (e.g., violence) should be explored in future studies based on an appropriate study population.

The current study discussed the long-term prognosis of PTSD symptoms based on information from mainly two time points (i.e., just after the disaster and 7 years after the disaster). Compared with previous studies on the short-term prognosis of PTSD symptoms^3,4^, the relationship between the risk factors and the predicted prognosis would be more complicated. Future studies that utilize information about new exposure after the disaster and detailed trajectory of PTSD symptoms would support our further understanding of the long-term prognosis of PTSD.

Although the LAMP minimizes false negatives by calibrating the Bonferroni factor, maintains statistical power under multiple comparisons and provides the significant *P* values for each combination against the outcomes, the risk factors identified by LAMP should be confirmed using ordinary statistical methods. In the current study, the validity of statistical methods was confirmed by checking the interaction, the distribution, and the variance explained by significant risk combinations as well as bivariate analysis for each risk component.

The current study has several limitations. First, the sample size was relatively small (624 subjects). This is a common problem for PTSD prognosis studies after natural disasters because a limited number of people are exposed to the disaster^39^. On the other hand, we achieved high levels of significance when we applied the combinational analysis, which suggests that the results in the present study are reliable. Second, the current MP-LAMP source code does not implement the function to adjust covariates. Therefore, we additionally performed the analysis using the target variable adjusted for potential confounding factors (Supplementary Table S3) and confirmed the consistency of the results. Considering the large overlap between significant risk combinations between the main analysis and the adjusted analysis, serious confounding was not observed in the current analysis. Third, each significant combination detected in the current study must be tested for reproducibility in an independent validation cohort in the future. To evaluate the generalizability of the results, future combinational risk studies conducted with different ethnicities or different traumatic experiences are needed.

## Conclusions

A comprehensive approach using MP-LAMP to detect significant combinations increased the power of the analysis and revealed significant risk combinations for high PTSD trajectory scores. Considering that hidden risk components were included in all of the detected significant risk combinations, a comprehensive combinational approach will be essential for detecting reliable risk combinations strongly associated with psychiatric conditions.

## Supplementary Information

Supplementary Information.

## Data Availability

The datasets analyzed during the current study are not publicly available due to ethical and privacy reasons.

## Abbreviations

PTSD: Posttraumatic stress disorder
MP-LAMP: Massive Parallel Limitless-Arity Multiple-testing Procedure
K6: Kessler Psychological Distress scale
AIS: Athens insomnia scale
LSNS-6: Lubben Social Network Scale-6
DSM: Diagnostic and Statistical Manual of Mental Disorders
IES-R: Impact of Event Scale-Revised
