# Supplementary material for: Machine learning to reveal hidden risk combinations for the trajectory of posttraumatic stress disorder symptoms
Source: Sci Rep. 2020 Dec 10;10:21726. doi: 10.1038/s41598-020-78966-z (PMC7730124; doi:10.1038/s41598-020-78966-z)
Supplement: Supplementary file 1 — Supplementary Information. [file 41598_2020_78966_MOESM1_ESM.pdf]

Supplementary Information

Machine learning to reveal hidden risk combinations for the trajectory of posttraumatic stress disorder symptoms

Yuta Takahashi, MD[a,b,c*], Kazuki Yoshizoe, PhD[d], Masao Ueki, PhD[b,d], Gen Tamiya, PhD[a,b,d], Yu Zhiqian, PhD[b,c], Yusuke Utsumi, MMSc[a], Atsushi Sakuma[a], MD, PhD, Koji Tsuda, PhD[d,e,] Atsushi Hozawa, MD, PhD[b], Ichiro Tsuji, MD, PhD[a,b], Hiroaki Tomita, MD, PhD[a,b,c*]

[a]Graduate School of Medicine, Tohoku University, 980-0872 Sendai, Japan

[b]Tohoku Medical Megabank Organization, Tohoku University, 980-8573 Sendai, Japan

[c]International Research Institute of Disaster Science, Tohoku University, 980-8572 Sendai, Japan

[d]RIKEN Center for Advanced Intelligence Project, 103-0027 Tokyo, Japan

[e]Department of Computational Biology and Medical Sciences, Graduate School of Frontier Sciences, The University of Tokyo, Chiba 277-8568, Japan

*__Corresponding author__

Yuta Takahashi

Email: yuta.takahashi@med.tohoku.ac.jp

Supplementary methods

Supplementary Tables

Supplementary Table S1: Significant risk combinations for PTSD trajectory scores detected by MP-LAMP.

Supplementary Table S2: The results of bivariate analyses for independent variables.

Supplementary Table S3: Significant risk combinations in the analysis adjusted for age and sex.

Supplementary Figure

Supplementary Figure S1: Boxplots of PTSD trajectory scores in the set of subjects selected by combinational or single risk factors

Supplementary methods.

Selecting testable combinations

Suppose that we have N subjects and that we know their PTSD trajectory score ranks. Given a risk combination, the set of subjects who have the risk combination is defined as J. N subjects are classified into $x = |J|$ risk and $N - x$ nonrisk subjects. Mann-Whitney U is defined as the probability that the ranks are more biased than J. The P-value is achieved at the smallest value when the PTSD trajectory scores in J are larger than the others or smaller than the others. The probability of the case appearing is described as

$$1/\binom{N}{x} \qquad (1)$$

This is the minimum P-value of Mann-Whitney U.

This value decreases with increasing x for $1 \leq x \leq N/2$ and takes the minimum value when $x = N/2$, which is a nonzero value.

Therefore, the smallest P-value depends on the number of all subjects and the number of subjects who have the risk combination. When x is small enough for the minimum P-value (equation (1)) to be larger than the significance level, the risk combination cannot be significant regardless of the PTSD trajectory scores of subjects.

Checking the validity of imputation process

We checked the validity of the imputation as follows according to the guidelines. [23]

First, we assessed the missing data before imputation by the following two steps.

1. The missing rates were calculated. The missing rates among IES-R items and potential risk factors were

0.5% and 2.9%, respectively. The highest missing rate for potential risk factors is 9.2%, which is about the current smoking rate.

2. We compared summary statistics (i.e., mean, median, the first quartile, the third quartile, minimum, maximum, and SD) of IES-R scores and potential risk factors between incomplete subjects and complete subjects, and we checked that there were no significant differences.

Second, after imputation, we assessed the validity of imputation as follows.

1. We performed an internal check for imputation.
   - The summary statistics (i.e., mean, median, the first quartile, the third quartile, minimum, maximum, and SD) of IES-R scores and potential risk factors between imputed datasets and complete datasets were compared.
   - The graphs of the distribution (i.e., histogram, density plot, quantile-quantile plot, and cumulative distribution plot) of imputed and complete datasets were compared.
2. We checked the imputation externally.
   - The distribution of imputed IES-R scores and other potential risk factors was compared with external Japanese datasets. [24] These external datasets were based on another Japanese disaster cohort study using a similar questionnaire as the current study. [24]

Based on the abovementioned procedure, we concluded that there would be no significant bias effect resulting from the imputation.

Converting the ordinary or continuous variables into binary variables

Among 61 potential risk factors, 53 risk factors were binary variables in the questionnaire and not converted.

Three scales (K6, AIS, and LSNS) were standard scales, and we followed previous studies for cutoff scores[25-28].

The remaining 5 risk factors were converted from ordinal (or continuous) scales into binary scales as follows. First, the walking time and resting time had three choices in the questionnaire. The two groups (highest and lowest categories) were analyzed as possible risk groups.

Walking time	<30 min	≥30 min, <1 h	≥1 h
Frequency	180	202	236
Short Walking time	○		
Long Walking time			○

Resting time	<3 h	≥3 h, <6 h	≥6 h
Frequency	387	153	72
Short resting time	○		
Long resting time			○

Second, for sleeping time and frequency of going out, the number of choices in the questionnaire was more than three.

Therefore, these items were discretized into ordinal variables with three levels of approximately equal frequency by using the infotheo R package and converted into two binary variables with the highest or lowest level as follows.

Sleeping time	<5 h	≥5 h, <6 h	≥6 h, <7 h	≥7 h, <8 h	≥8 h, <9 h	≥9 h
Frequency	56	204	196	113	39	14
Short sleeping time	○	○				
Long sleeping time				○	○	○

Frequency of going out	Very little	Approximately 1 day/month	Approximately 1 day/week	Approximately 3 day/week	About Every day
Frequency	18	10	49	124	418
Low frequency of going out	○	○	○		
High frequency of going out					○

Finally, age is a continuous variable. This variable was divided into three groups of approximately similar frequency. Then, we prepared not only the youngest and oldest but also the middle age group because of the possibility of a nonlinear relationship between age and psychiatric symptoms.[1]

Age	<30	30-39	40-49	50-59	60-69	70-79	80-
Frequency	63	74	108	109	151	103	16
Younger age	○	○	○				
Middle age				○	○		
Older age						○	○

For single components included in significant risk combinations, to minimize bias, the conventional analysis was performed (i.e., checking distribution of original ordinal scale and the consistency of the results by setting various cutoffs).

Supplementary Table S1. Significant risk combinations for PTSD trajectory scores detected by MP-LAMP.

**Significant combinations**	**Detected interactions (P<0.05)**	**Raw P-value[a]**	**Adjusted P-value[a]**	**Frequency**	**Variance explained (%)**
Unemployment (A), Short walking time (≤30min/d) (B), Short resting time (sitting/napping <3h/d) (C), Evacuation without preparation (D)	B*C, C*D, A*B*C	3.1×10^{-9}	2.0×10^{-4}	29	6.4
Female (A), Short walking time (≤30min/d) (B), Short resting time (sitting/napping <3h/d) (C), Evacuation without preparation (D), Decreased income (E), Decreased volume of work (F)	B*C, C*D, A*E, C*F, C*D*F, B*D*E*F	1.2×10^{-8}	8.1×10^{-4}	17	7.2
Female (A), Short walking time (≤30min/d) (B), Short resting time (sitting/napping <3h/d) (C), Evacuation without preparation (D), Decreased volume of work (E)	B*C, C*D, A*E, A*C*E, C*D*E	1.4×10^{-8}	9.3×10^{-4}	18	6.8
Female (A), Short resting time (sitting/napping <3h/d) (B), Evacuation without preparation (C), Decreased volume of work (D)	B*C, A*D, B*C*D	1.5×10^{-8}	1.0×10^{-3}	45	5.9
Unemployment (A), Short walking time (≤30min/d) (B), Short resting time (sitting/napping <3h/d) (C), Evacuation without preparation (D), Life-threatening experience (E)	B*C, C*D, A*B*C	1.8×10^{-8}	1.2×10^{-3}	19	7.3
Female (A), Short resting time (sitting/napping <3h/d) (B), Evacuation without preparation (C), Decreased income (D), Decreased volume of work (E)	B*C, A*D, B*E, B*C*E	1.8×10^{-8}	1.2×10^{-3}	44	6.0
Female (A), Short resting time (sitting/napping <3h/d) (B), Evacuation without preparation (C), Decreased income (D), Decreased volume of work (E), K6 ≥5 (F)	B*C, A*D, B*E, B*C*E, A*C*E*F, B*C*E*F	3.0×10^{-8}	2.0×10^{-3}	36	6.0
Unemployment (A), Short resting time (sitting/napping <3h/d) (B), Evacuation without preparation (C), Life-threatening experience (D), Decreased income (E), Decreased volume of work (F)	B*C, B*F, A*D*E, B*C*F, A*B*C*E, A*B*C*F	4.6×10^{-8}	3.0×10^{-3}	15	8.3

Significant combinations	Detected interactions (P<0.05)	Raw P-value[a]	Adjusted P-value[a]	Frequency	Variance explained (%)
Female (A), Short walking time (≤30min/d) (B), Short resting time (sitting/napping <3h/d) (C), Evacuation without preparation (D), Decreased income (E), Decreased volume of work (F), K6 ≥5 (G)	B*C, C*D, A*E, C*F, C*D*F, A*D*F*G, C*D*F*G, A*B*C*D*G, B*C*D*E*G, A*B*C*D*E*G	5.0×10^{-8}	3.3×10^{-3}	16	6.0
Unemployment (A), Short resting time (sitting/napping <3h/d) (B), Evacuation without preparation (C), Life-threatening experience (D), Decreased volume of work (E)	B*C, B*C*E, A*D*E, A*B*C*E	5.4×10^{-8}	3.6×10^{-3}	16	7.8
Female (A), Unemployment (B), Short walking time (≤30min/d) (C), Short resting time (sitting/napping <3h/d) (D), Evacuation without preparation (E)	C*D, D*E, B*C*D	6.1×10^{-8}	4.1×10^{-3}	23	5.1
Unemployment (A), Short walking time (≤30min/d) (B), Short resting time (sitting/napping <3h/d) (C), Evacuation without preparation (D), K6 ≥5 (E)	B*C, C*D, B*E, A*B*C	1.0×10^{-7}	7.0×10^{-3}	19	5.7
Female (A), AIS ≥6 (B), Short resting time (sitting/napping <3h/d) (C), Evacuation without preparation (D), Decreased income (E), Decreased volume of work (F)	C*D, A*E, C*F, C*D*F, B*C*D*F	1.2×10^{-7}	8.6×10^{-3}	29	4.3
Female (A), Short walking time (≤30min/d) (B), Evacuation without preparation (C), Decreased income (D), Decreased volume of work (E), K6 ≥5 (F)	A*D, B*F, A*C*E*F	1.2×10^{-7}	8.6×10^{-3}	26	4.8
Female (A), Older age (≥65) (B), Short resting time (sitting/napping <3h/d) (C), Evacuation without preparation (D), Decreased volume of work (E)	A*B, C*D, A*B*C, B*C*D, C*D*E	1.3×10^{-7}	8.7×10^{-3}	27	5.0
Female (A), Short walking time (≤30min/d) (B), Short resting time (sitting/napping <3h/d) (C), Evacuation without preparation (D), Decreased income (E)	B*C, C*D, A*E	1.3×10^{-7}	8.8×10^{-3}	23	6.0
Female (A), Unemployment (B), Short walking time (≤30min/d) (C), Short resting time (sitting/napping <3h/d) (D)	C*D, B*C*D	1.3×10^{-7}	9.2×10^{-3}	39	4.4

**Significant combinations**	**Detected interactions (P<0.05)**	**Raw P-value[a]**	**Adjusted P-value[a]**	**Frequency**	**Variance explained (%)**
Unemployment (A), Short resting time (sitting/napping <3h/d) (B), Evacuation without preparation (C), Decreased volume of work (D)	B*C, B*C*D, A*B*C*D	1.4×10^{-7}	9.3×10^{-3}	28	6.1
Female (A), Older age (≥65) (B), Short resting time (sitting/napping <3h/d) (C), Evacuation without preparation (D), Decreased income (E), Decreased volume of work (F)	A*B, C*D, A*E, C*F, A*B*C, B*C*D, C*D*F, B*E*F	1.4×10^{-7}	9.4×10^{-3}	26	5.2
Unemployment (A), Short walking time (≤30min/d) (B), Short resting time (sitting/napping <3h/d) (C), Evacuation without preparation (D), Life-threatening experience (E), Decreased income (F)	B*C, C*D, A*B*C, A*E*F, A*C*D*F	1.5×10^{-7}	0.010	12	8.5
Unemployment (A), Short resting time (sitting/napping <3h/d) (B), Evacuation without preparation (C), Decreased income (D), Decreased volume of work (E)	B*C, B*E, B*C*E, A*B*C*D, A*B*C*E	1.5×10^{-7}	0.010	27	6.3
Short walking time (≤30min/d) (A), Short resting time (sitting/napping <3h/d) (B), Evacuation without preparation (C), Intense fear, hopelessness, and horror (D), K6 ≥5 (E)	A*B, B*C, A*E, D*E, A*C*D*E	1.8×10^{-7}	0.012	35	6.4
Unemployment (A), Short walking time (≤30min/d) (B), Short resting time (sitting/napping <3h/d) (C)	B*C, A*B*C	1.8×10^{-7}	0.012	49	4.4
Unemployment (A), Short resting time (sitting/napping <3h/d) (B), Evacuation without preparation (C), Life-threatening experience (D), Decreased income (E), Decreased volume of work (F), K6 ≥5 (G)	B*C, B*F, A*D*E, B*C*F, A*B*C*E, B*C*F*G	1.9×10^{-7}	0.013	14	7.0
Female (A), Unemployment (B), Short resting time (sitting/napping <3h/d) (C), Evacuation without preparation (D), Decreased volume of work (E)	C*D, A*E, C*D*E, B*C*D*E	2.0×10^{-7}	0.013	24	5.4
Short walking time (≤30min/d) (A), Short resting time (sitting/napping <3h/d) (B), Evacuation without preparation (C)	A*B, B*C	2.0×10^{-7}	0.013	81	5.5

Significant combinations	Detected interactions (P<0.05)	Raw P-value[a]	Adjusted P-value[a]	Frequency	Variance explained (%)
Female (A), Unemployment (B), Short resting time (sitting/napping <3h/d) (C), Evacuation without preparation (D), Life-threatening experience (E), Decreased income (F), Decreased volume of work (G)	C*D, A*F, C*G, B*E*F, C*D*G, B*C*D*F, B*C*D*G	2.0×10^{-7}	0.013	14	6.5
Female (A), Unemployment (B), Short resting time (sitting/napping <3h/d) (C), Evacuation without preparation (D), Decreased income (E), Decreased volume of work (F)	C*D, A*E, C*F, C*D*F	2.1×10^{-7}	0.014	23	5.6
Unemployment (A), Short walking time (≤30min/d) (B), Short resting time (sitting/napping <3h/d) (C), Evacuation without preparation (D), Loss of family/friends (E)	B*C, C*D, D*E, A*B*C, A*B*E	2.1×10^{-7}	0.014	14	6.6
Female (A), Unemployment (B), Short resting time (sitting/napping <3h/d) (C), Evacuation without preparation (D), Life-threatening experience (E), Decreased volume of work (F)	C*D, A*F, C*D*F, B*E*F, B*C*D*F	2.4×10^{-7}	0.016	15	6.1
Female (A), Physical condition (not good) (B), Short resting time (sitting/napping <3h/d) (C), Evacuation without preparation (D), Decreased income (E), Decreased volume of work (F), K6 ≥5 (G)	A*B, C*D, A*E, C*F, A*B*D, A*B*E, B*C*E, C*D*F, B*D*G, B*C*D*F, B*D*E*F, A*B*D*G, B*C*F*G, A*D*F*G	3.1×10^{-7}	0.021	14	5.4
Female (A), AIS ≥6 (B), Short resting time (sitting/napping <3h/d) (C), Evacuation without preparation (D), Decreased income (E), Decreased volume of work (F), K6 ≥5 (G)	C*D, A*E, C*F, C*D*F, A*F*G, B*C*D*F, A*D*F*G	3.2×10^{-7}	0.022	27	4.3
Female (A), Short resting time (sitting/napping <3h/d) (B), Evacuation without preparation (C), Intense fear, hopelessness, and horror (D), Decreased income (E), Decreased volume of work (F), K6 ≥5 (G)	B*C, A*E, B*F, D*G, B*C*F, B*D*F, A*D*E*G, A*C*F*G, B*C*F*G	3.7×10^{-7}	0.024	27	4.7

**Significant combinations**	**Detected interactions (P<0.05)**	**Raw P-value[a]**	**Adjusted P-value[a]**	**Frequency**	**Variance explained (%)**
Female (A), Short walking time (≤30min/d) (B), Short resting time (sitting/napping <3h/d) (C), Decreased volume of work (D)	B*C, A*D, C*D, A*C*D	4.3×10^{-7}	0.028	26	5.1
Female (A), Short walking time (≤30min/d) (B), Evacuation without preparation (C), Loss of family/friends (D), Decreased income (E), Decreased volume of work (F)	C*D, A*E, A*B*D*F	4.4×10^{-7}	0.029	16	6.3
Female (A), Short sleeping time (<6h/d) (B), Short walking time (≤30min/d) (C), Evacuation without preparation (D), K6 ≥5 (E)	None	4.4×10^{-7}	0.029	26	3.3
Unemployment (A), Short walking time (≤30min/d) (B), Short resting time (sitting/napping <3h/d) (C), Evacuation without preparation (D), Intense fear, hopelessness, and horror (E)	B*C, C*D, A*B*C	4.4×10^{-7}	0.029	19	4.7
Female (A), AIS ≥6 (B), Short walking time (≤30min/d) (C), Evacuation without preparation (D), Decreased income (E), K6 ≥5 (F)	A*E, C*F	4.5×10^{-7}	0.030	20	2.9
Female (A), Unemployment (B), Short walking time (≤30min/d) (C), Short resting time (sitting/napping <3h/d) (D), Evacuation without preparation (E), Life-threatening experience (F)	C*D, D*E, B*C*D, C*E*F, A*C*D*E*F	4.6×10^{-7}	0.030	16	5.3
Short walking time (≤30min/d) (A), Evacuation without preparation (B), Intense fear, hopelessness, and horror (C), K6 ≥5 (D)	A*D, C*D, A*B*C*D	4.9×10^{-7}	0.032	54	4.6
Unemployment (A), Short resting time (sitting/napping <3h/d) (B), Evacuation without preparation (C), Life-threatening experience (D), Decreased income (E)	B*C, A*D*E, A*B*C*E	5.0×10^{-7}	0.033	20	6.8
Unemployment (A), Short walking time (≤30min/d) (B), Short resting time (sitting/napping <3h/d) (C), Evacuation without preparation (D), Life-threatening experience (E), K6 ≥5 (F)	B*C, C*D, B*F, A*B*C	5.4×10^{-7}	0.036	12	6.5

Significant combinations	Detected interactions (P<0.05)	Raw P-value[a]	Adjusted P-value[a]	Frequency	Variance explained (%)
Female (A), Short walking time (≤30min/d) (B), Short resting time (sitting/napping <3h/d) (C), Evacuation without preparation (D), Life-threatening experience (E), Decreased income (F), Decreased volume of work (G)	B*C, C*D, A*F, C*G, B*D*E, C*D*G	5.4×10^{-7}	0.036	12	6.2
Unemployment (A), Short walking time (≤30min/d) (B), Short resting time (sitting/napping <3h/d) (C), Evacuation without preparation (D), Decreased income (E)	B*C, C*D, A*B*C, A*C*D*E	5.5×10^{-7}	0.036	15	7.1
Unemployment (A), Short resting time (sitting/napping <3h/d) (B), Evacuation without preparation (C), Life-threatening experience (D), Loss of family/friends (E), Decreased income (F), Decreased volume of work (G)	B*C, C*E, B*G, C*D*E, A*D*F, B*C*G, E*F*G, A*B*C*F, B*C*D*F, B*C*E*F, A*B*C*D*G	5.5×10^{-7}	0.036	12	8.0
Short walking time (≤30min/d) (A), Short resting time (sitting/napping <3h/d) (B), Evacuation without preparation (C), Intense fear, hopelessness, and horror (D)	A*B, B*C	5.5×10^{-7}	0.037	52	4.9
Older age (≥65) (A), Short walking time (≤30min/d) (B), Short resting time (sitting/napping <3h/d) (C), Evacuation without preparation (D)	B*C, C*D	6.2×10^{-7}	0.041	32	4.6
Unemployment (A), Short resting time (sitting/napping <3h/d) (B), Evacuation without preparation (C), Life-threatening experience (D), Loss of family/friends (E), Decreased income (F)	B*C, C*E, C*D*E, A*B*F, A*D*F, A*B*C*F, A*C*D*E*F	6.3×10^{-7}	0.042	13	7.5
Female (A), AIS ≥6 (B), Short walking time (≤30min/d) (C), Evacuation without preparation (D), Decreased income (E), Decreased volume of work (F), K6 ≥5 (G)	A*E, C*G, E*F*G, A*D*F*G	6.5×10^{-7}	0.043	18	3.0
Female (A), Short walking time (≤30min/d) (B), Short resting time (sitting/napping <3h/d) (C), Evacuation without preparation (D), Life-threatening experience (E), Decreased volume of work (F)	B*C, C*D, A*F, A*C*F, C*D*F	6.6×10^{-7}	0.044	13	5.8

Significant combinations	Detected interactions (P<0.05)	Raw P-value[a]	Adjusted P-value[a]	Frequency	Variance explained (%)
Short resting time (sitting/napping <3h/d) (A), Evacuation without preparation (B), Decreased volume of work (C)	A*B, A*B*C	6.6×10^{-7}	0.044	83	5.6
Female (A), Unemployment (B), Short walking time (≤30min/d) (C), Short resting time (sitting/napping <3h/d) (D), Evacuation without preparation (E), Life-threatening experience (F), Decreased income (G)	C*D, D*E, A*G, B*C*D, C*E*F, B*F*G, B*D*E*G, A*C*D*E*F, B*C*D*F*G	6.9×10^{-7}	0.046	11	6.6
Older age (≥65) (A), Short walking time (≤30min/d) (B), Short resting time (sitting/napping <3h/d) (C), Evacuation without preparation (D), Decreased income (E), Decreased volume of work (F), K6 ≥5 (G)	B*C, C*D, B*G, C*D*F, B*D*E*G, A*B*C*D*E	7.0×10^{-7}	0.046	16	5.1
Older age (≥65) (A), Unemployment (B), Short walking time (≤30min/d) (C), Short resting time (sitting/napping <3h/d) (D), Evacuation without preparation (E), Life-threatening experience (F)	C*D, D*E, A*D*E	7.0×10^{-7}	0.047	13	6.2
Short walking time (≤30min/d) (A), Short resting time (sitting/napping <3h/d) (B), Evacuation without preparation (C), K6 ≥5 (D)	A*B, B*C, A*D	7.3×10^{-7}	0.049	53	5.1
Unemployment (A), Short walking time (≤30min/d) (B), Short resting time (sitting/napping <3h/d) (C), Evacuation without preparation (D), Decreased income (E), Decreased volume of work (F)	B*C, C*D, C*F, A*B*C, A*B*F, C*D*F, A*C*D*E, A*C*D*F	7.4×10^{-7}	0.049	12	7.2

[a] P-values were calculated based on the Mann-Whitney U test using the PTSD trajectory score as a response variable.

Abbreviations: PTSD, posttraumatic stress disorder; MP-LAMP, Massive Parallel Limitless-Arity Multiple-testing Procedure

Supplementary Table S2. The results of bivariate analyses for independent variables.

**Variables**	**Average PTSD trajectory score in selected group**	**Average PTSD trajectory score in unselected group**	**Frequency**	**Raw P-value**[a]	**Adjusted P-value**[b]	**Variance explained (%)**	**Significant combinations**
Older age (≥65)	2.18	-0.98	194	2.5×10^{-3}	0.150	1.4	Included
Injured	4.26	-0.56	73	3.6×10^{-3}	0.218	1.6	Not included
PMH of hypertension	3.45	-0.92	131	0.010	0.581	1.9	Not included
Swept by tsunami	6.51	-0.25	23	0.010	0.606	1.1	Not included
PMH of diabetes mellitus	5.58	-0.41	43	0.011	0.660	1.5	Not included
PMH of myocardial infarction	7.43	-0.27	22	0.014	0.864	1.3	Not included
Short walking time (≤30min/d)	1.62	-1.00	238	0.017	>1	1.1	Included
Life-threatening experience	0.95	-1.15	342	0.019	>1	0.7	Included
Decreased volume of work	2.47	-1.22	207	0.021	>1	2.0	Included
Physical condition (not good)	2.37	-0.56	120	0.033	>1	0.9	Included
Evacuation without preparation	0.90	-1.41	380	0.044	>1	0.8	Included
Loss of children	13.85	-0.07	3	0.048	>1	0.6	Not included
Short resting time (sitting/napping <3h/d)	0.68	-1.21	399	0.055	>1	0.5	Included
PMH of arthritis	5.41	-0.23	25	0.083	>1	0.8	Not included
Occupation: education and medical welfare	-3.88	0.24	36	0.087	>1	0.6	Not included
PMH of allergic diseases	-4.13	0.17	25	0.106	>1	0.4	Not included
Decreased income	1.47	-1.24	285	0.130	>1	1.2	Included
PMH of dental diseases	2.89	-0.35	68	0.139	>1	0.7	Not included
Physical condition (poor)	5.97	-0.11	11	0.150	>1	2.1	Not included
Occupation: postal services	1.58	-0.10	37	0.161	>1	0.1	Not included
PMH of stroke	7.88	-0.08	6	0.165	>1	0.4	Not included
Low frequency of going out (≤3d/w)	0.32	-0.15	201	0.199	>1	0.3	Not included

Variables	Average PTSD trajectory score in selected group	Average PTSD trajectory score in unselected group	Frequency	Raw P-value[a]	Adjusted P-value[b]	Variance explained (%)	Significant combinations
PMH of other diseases	2.26	-0.25	63	0.225	>1	0.3	Not included
PMH of cancer	4.84	-0.11	14	0.226	>1	0.3	Not included
PMH of incurable diseases specified	4.37	-0.06	8	0.259	>1	0.1	Not included
PMH of lung diseases	-2.89	0.10	21	0.266	>1	0.2	Not included
Short sleeping time (<6h/d)	0.52	-0.38	262	0.279	>1	0.1	Included
Occupation: agriculture	3.58	-0.17	28	0.310	>1	0.4	Not included
PMH of liver diseases	-3.54	0.04	7	0.333	>1	0.0	Not included
PMH of depression	4.18	-0.03	5	0.339	>1	0.1	Not included
Loss of brothers/sisters	-1.34	0.02	11	0.393	>1	0.0	Not included
Witnessing other people's death	0.39	-0.09	116	0.411	>1	0.0	Not included
Unemployment	1.26	-0.70	222	0.440	>1	0.6	Included
Female	0.34	-0.46	358	0.454	>1	0.1	Included
PMH of dyslipidemia	2.19	-0.30	74	0.457	>1	0.0	Not included
Intense fear, hopelessness, and horror	0.51	-0.69	359	0.459	>1	0.2	Included
PMH of osteoporosis	-0.02	0.00	16	0.463	>1	0.0	Not included
Occupation: finance and insurance industry	-3.55	0.03	6	0.471	>1	0.0	Not included
PMH of anemia	-4.26	0.04	6	0.485	>1	0.0	Not included
Occupation: service industry	-1.56	0.14	52	0.496	>1	0.0	Not included
Witnessing tsunami	0.44	-0.48	323	0.515	>1	0.0	Not included
AIS ≥6	0.60	-0.41	253	0.525	>1	0.1	Included
Occupation: information and communication	-5.16	0.03	4	0.532	>1	0.0	Not included
Loss of relatives	-0.15	0.06	171	0.560	>1	0.0	Not included
K6 ≥5	0.29	-0.29	314	0.567	>1	0.0	Included
Loss of family/friends	0.55	-0.50	298	0.581	>1	0.2	Included
Occupation: fishing	-0.49	0.05	56	0.636	>1	0.0	Not included

Variables	Average PTSD trajectory score in selected group	Average PTSD trajectory score in unselected group	Frequency	Raw P-value[a]	Adjusted P-value[b]	Variance explained (%)	Significant combinations
PMH of gastroduodenal ulcer	1.86	-0.05	17	0.640	>1	0.0	Not included
K6 ≥13	1.00	-0.07	39	0.681	>1	0.0	Not included
Other jobs	0.34	-0.04	59	0.696	>1	0.0	Not included
Occupation: retail industry	-0.84	0.07	51	0.705	>1	0.0	Not included
Loss of friends	0.78	-0.11	79	0.753	>1	0.0	Not included
Occupation: manufacturing industry	-0.98	0.09	51	0.789	>1	0.0	Not included
Occupation: electricity, gas, and water	0.25	0.00	9	0.801	>1	0.0	Not included
Current alcohol consumption	-0.07	0.03	189	0.802	>1	0.0	Not included
Current smoking	-1.15	0.34	144	0.806	>1	0.0	Not included
Occupation: construction industry	-0.07	0.00	34	0.815	>1	0.0	Not included
Loss of coworkers	0.46	-0.01	17	0.871	>1	0.0	Not included
PMH of kidney diseases	0.01	0.00	7	0.877	>1	0.0	Not included
LSNS <12	0.04	-0.01	123	0.897	>1	0.0	Not included

The PTSD trajectory score is the IES-R scores after 8 years adjusted for the baseline score.

[a] P-values were calculated using a linear regression model with the PTSD trajectory score as a response variable and adjusted by age and gender.

[b] Multiple comparisons were subjected to the Bonferroni correction to control the familywise error rate.

Abbreviations: PTSD, posttraumatic stress disorder; PMH, past medical history; AIS, Athens Insomnia Scale; K6, Kessler Psychological Distress Scale; LSNS-6, Lubben Social Network Scale-6

Supplementary Table S3. Significant risk combinations in the analysis adjusted for age and sex.

**Significant risk combinations**	**Raw P-value[a]**	**Adjusted P-value[a]**	**Frequency**	**Significant combinations in the main analysis[b]**
Female; Short walking time (≤30min/d); Short resting time (sitting/napping <3h/d); Evacuation without preparation; Decreased income; Decreased volume of work	7.7×10^{-8}	5.1×10^{-3}	17	Included
Unemployment; Short walking time (≤30min/d); Short resting time (sitting/napping <3h/d); Evacuation without preparation; Life-threatening experience	1.0×10^{-7}	6.8×10^{-3}	19	Included
Unemployment; Short resting time (sitting/napping <3h/d); Evacuation without preparation; Life-threatening experience; Decreased income; Decreased volume of work	1.0×10^{-7}	7.3×10^{-3}	15	Included
Unemployment; Short walking time (≤30min/d); Short resting time (sitting/napping <3h/d); Evacuation without preparation	1.2×10^{-7}	8.2×10^{-3}	29	Included
Female; Short walking time (≤30min/d); Short resting time (sitting/napping <3h/d); Evacuation without preparation; Decreased volume of work	1.3×10^{-7}	8.7×10^{-3}	18	Included
Unemployment; Short resting time (sitting/napping <3h/d); Evacuation without preparation; Life-threatening experience; Decreased volume of work	1.9×10^{-7}	1.3×10^{-2}	16	Included
Short walking time (≤30min/d); Short resting time (sitting/napping <3h/d); Evacuation without preparation	2.6×10^{-7}	1.7×10^{-2}	81	Included
Female; Short resting time (sitting/napping <3h/d); Evacuation without preparation; Decreased income; Decreased volume of work	2.6×10^{-7}	1.7×10^{-2}	44	Included

**Significant risk combinations**	**Raw P-value[a]**	**Adjusted P-value[a]**	**Frequency**	**Significant combinations in the main analysis[b]**
Unemployment; Short walking time (≤30min/d); Short resting time (sitting/napping <3h/d); Evacuation without preparation; Life-threatening experience; Decreased income	2.8×10^{-7}	1.8×10^{-2}	12	Included
Female; Short resting time (sitting/napping <3h/d); Evacuation without preparation; Decreased volume of work	2.9×10^{-7}	1.9×10^{-2}	45	Included
Female; Short walking time (≤30min/d); Short resting time (sitting/napping <3h/d); Evacuation without preparation; Decreased income; Decreased volume of work; K6 ≥5	3.0×10^{-7}	2.0×10^{-2}	16	Included
Unemployment; Short resting time (sitting/napping <3h/d); Evacuation without preparation; Life-threatening experience; Decreased income; Decreased volume of work; K6 ≥5	4.4×10^{-7}	2.9×10^{-2}	14	Included
Female; Short resting time (sitting/napping <3h/d); Evacuation without preparation; Decreased income; Decreased volume of work; K6 ≥5	4.5×10^{-7}	3.0×10^{-2}	36	Included
Female; Unemployment; Short resting time (sitting/napping <3h/d); Evacuation without preparation; Life-threatening experience; Decreased income; Decreased volume of work	4.7×10^{-7}	3.2×10^{-2}	14	Included
Short walking time (≤30min/d); Short resting time (sitting/napping <3h/d); Evacuation without preparation; Intense fear, hopeless, and horror	5.3×10^{-7}	3.6×10^{-2}	14	Included

[a] P-values were calculated based on the Mann-Whitney U test using the PTSD trajectory score adjusted for sex and age as a response variable.

[b] This column indicates whether the combinations were included in the significant risk combinations in the main analysis, which is not adjusted for sex and age.

Supplementary Figure S1

Boxplots of PTSD trajectory scores in the set of subjects selected by combinational or single risk factors.

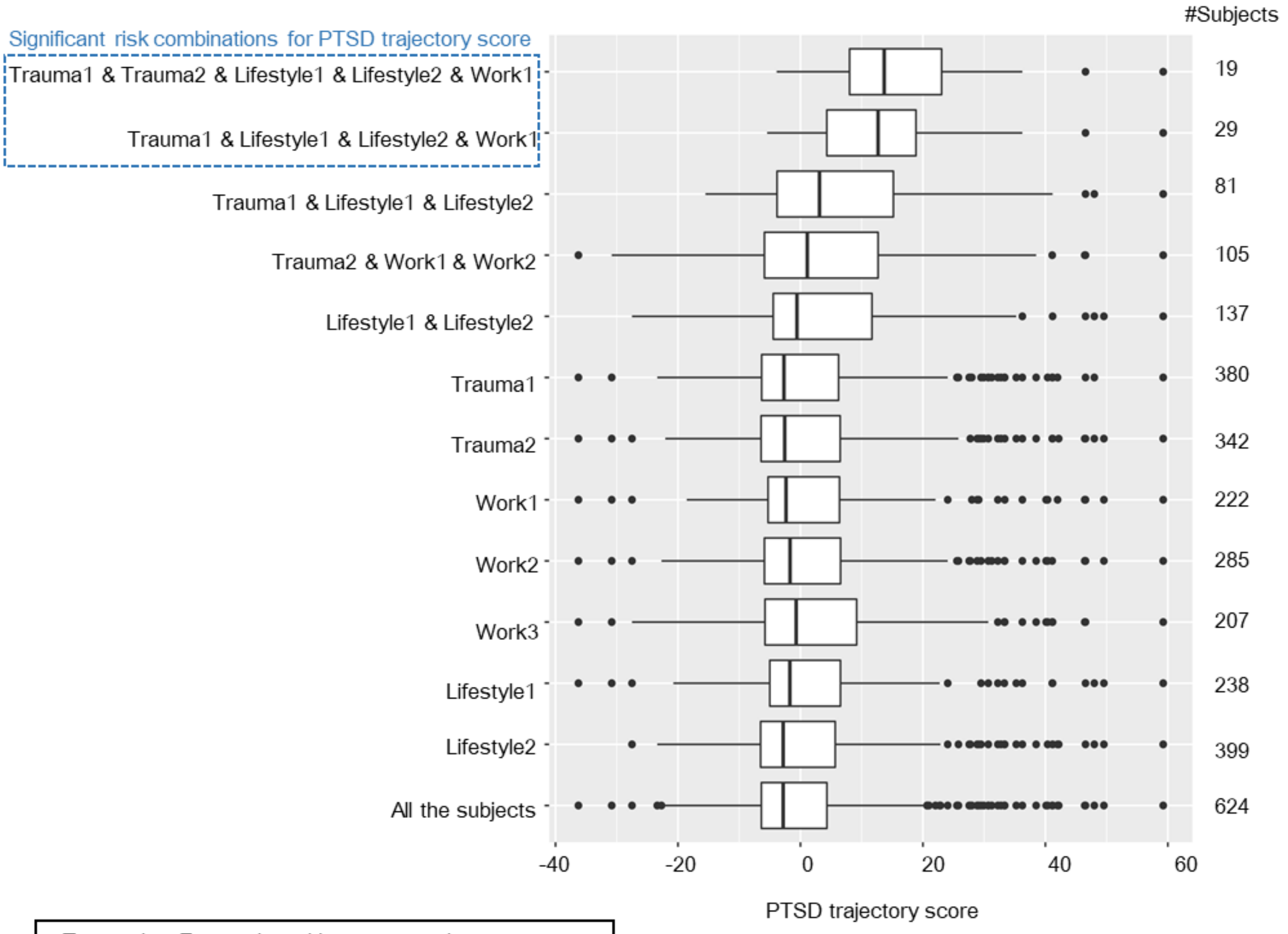

Trauma1	Evacuation without preparation
Trauma2	Life-threatening experience
Work1	Unemployment
Work2	Decreased income
Work3	Decreased volume of work
Lifestyle1	Short working time (≤ 30 min/d)
Lifestyle2	Short resting time (sitting/napping ≤ 3 h/d)

Referenece

1 Nguyen, C. D., Carlin, J. B. & Lee, K. J. Model checking in multiple imputation: an overview and case study. *Emerging Themes in Epidemiology* **14**, 8, doi:10.1186/s12982-017-0062-6 (2017).

2 Kuriyama, S. *et al.* The Tohoku Medical Megabank Project: Design and Mission. *Journal of epidemiology* **26**, 493-511, doi:10.2188/jea.JE20150268 (2016).

3 Prochaska, J. J., Sung, H. Y., Max, W., Shi, Y. & Ong, M. Validity study of the K6 scale as a measure of moderate mental distress based on mental health treatment need and utilization. *International journal of methods in psychiatric research* **21**, 88-97, doi:10.1002/mpr.1349 (2012).

4 Furukawa, T. A., Kessler, R. C., Slade, T. & Andrews, G. The performance of the K6 and K10 screening scales for psychological distress in the Australian National Survey of Mental Health and Well-Being. *Psychol Med* **33**, 357-362 (2003).

5 Soldatos, C. R., Dikeos, D. G. & Paparrigopoulos, T. J. The diagnostic validity of the Athens Insomnia Scale. *Journal of psychosomatic research* **55**, 263-267 (2003).

6 Lubben, J. *et al.* Performance of an abbreviated version of the Lubben Social Network Scale among three European community-dwelling older adult populations. *The Gerontologist* **46**, 503-513 (2006).

7 Kessler, R. C. *et al.* Trends in mental illness and suicidality after Hurricane Katrina. *Mol Psychiatry* **13**, 374-384, doi:10.1038/sj.mp.4002119 (2008).
